# *Propionibacterium freudenreichii* Inhibits RANKL-Induced Osteoclast Differentiation and Ameliorates Rheumatoid Arthritis in Collagen-Induced Arthritis Mice

**DOI:** 10.3390/microorganisms10010048

**Published:** 2021-12-27

**Authors:** Jiah Yeom, Dong Joon Yim, Seongho Ma, Young-Hee Lim

**Affiliations:** 1 Department of Integrated Biomedical and Life Sciences, Graduate School, Korea University, Seoul 02841, Korea; intro56@naver.com (J.Y.); abmatics@naver.com (D.J.Y.); aktjdgh8@naver.com (S.M.); 2 School of Biosystems and Biomedical Sciences, College of Health Science, Korea University, Seoul 02841, Korea; 3 Department of Laboratory Medicine, Korea University Guro Hospital, Seoul 08308, Korea

**Keywords:** rheumatoid arthritis, *Propionibacterium freudenreichii*, osteoclast, collagen-induced arthritis

## Abstract

Osteoclast differentiation is crucial for bone absorption, and osteoclasts are involved in bone destruction in rheumatoid arthritis (RA). Dairy *Propionibacterium freudenreichii* is used as a cheese starter and possesses prebiotic and postbiotic properties. It is known to stimulate the growth of bifidobacteria and produces valuable metabolites, such as vitamin B12 and propionic acid. However, limited information is available on the beneficial effects of *P. freudenreichii* on human disease. Herein, we aimed to investigate the inhibitory effect of *P. freudenreichii* MJ2 (MJ2) isolated from raw milk on osteoclast differentiation and evaluate the improvement in RA. The murine macrophage cell line, RAW 264.7, and a collagen-induced arthritis (CIA) mouse model were used to perform in vitro and in vivo studies, respectively. Heat-killed *P. freudenreichii* MJ2 (hkMJ2)-treated cells significantly inhibited RANKL-induced osteoclast differentiation and TRAP activity. HkMJ2-treated cells exhibited significantly decreased expression of genes and proteins related to RANKL-induced osteoclast differentiation. MJ2 administration decreased the arthritic score in the CIA mouse model. Live and dead MJ2 inhibited bone loss and afforded protection against bone erosion and joint damage in CIA mice. MJ2 decreased the levels of collagen-specific antibodies and inflammatory cytokines and the expression of osteoclast differentiation-related genes and proteins in CIA mice. Interestingly, live and dead MJ2 showed similar RA improvement effects in CIA mice. In conclusion, *P. freudenreichii* MJ2 inhibited osteoclast differentiation by inhibiting the *NF-κB* signaling pathway and ameliorated CIA.

## 1. Introduction

Rheumatoid arthritis (RA) is a common disease that occurs in a large proportion of the global population and affects females more than males [1]. RA is an autoimmune disorder that attacks the body’s defense system and primarily causes inflammation in the joints of the knee or fingers. In addition, RA affects other organs, including the eyes, lungs, and heart, and RA-induced chronic inflammation can impact bone absorption and osteolysis [2]. Currently, no available drug can completely cure RA. However, some drugs such as steroids, nonsteroidal anti-inflammatory drugs (NSAIDs), and disease-modifying anti-rheumatic drugs (DMARDs) can delay the progression of RA or relieve pain and inflammation; however, patients taking drugs such as aspirin, antidepressants, and antihypertensives might be at a higher risk for additive effects when combined with these drugs. In addition, NSAIDs and DMARDs are known to induce side effects, such as liver damage, infection, and induction of tuberculosis [3,4]. Therefore, the development of effective and safe therapeutic agents is necessary to combat RA.

RA-induced symptoms include joint swelling, pain, and joint stiffness [2]. Since the most typical symptoms of RA are inflammation and pain, the studies for developing treatment drugs have focused mainly on reducing inflammation status [3,5]. Recently, blocking the activity of osteoclasts that absorb bone structure is now receiving attention. In RA patients’ synovium, angiogenesis and bone turnover toward osteoclasts are increased [6]. The inflow of pro-inflammatory cytokines and chemokines secreted from osteoclasts recruit more osteoclast precursors and then eventually inflammation and bone destruction are getting worse with the RA process [7]. In addition, the microenvironments in RA produce a suitable condition that facilitates osteoclasts differentiation from macrophages by increasing the receptor activator of nuclear factor-κB ligand (RANKL)/osteoprotegerin (*OPG*) ratio [8]. Therefore, it is important to prevent osteoclast differentiation and bone loss for the treatment of RA.

The *OPG*/RANKL/receptor activator of nuclear factor κ (*RANK*) system between osteoblasts and osteoclasts has gained momentum as a new target to alleviate bone-related diseases such as RA and osteoporosis [8]. *RANKL* is a member of the tumor necrosis factor (TNF) superfamily and induces osteoclast differentiation by binding to *RANK*, which is expressed on osteoclast precursors. *OPG* is a soluble decoy receptor bound to *RANKL* and blocks the RANKL-RANK interaction, consequently inhibiting osteoclast differentiation [9]. Therefore, the *OPG*/*RANKL*/*RANK* system could be a potential target for treating bone-related diseases. Specifically, as RA-induced *RANKL* expression is a major factor in joint destruction, chemical or biological agents capable of inhibiting *RANKL* expression have been developed to prevent joint destruction in patients with RA [10]. However, for adequately treating RA, most drugs primarily focus on attenuating inflammation, and only a few agents target inhibition of osteoclast differentiation, especially inhibition of the RANKL-RANK interaction. Denosumab, a fully human monoclonal anti-RANKL antibody, prevents bone erosion in RA and inhibits osteoclast differentiation and bone absorption by blocking RANKL-RANK binding [11]. Treatment with *OPG* afforded a preventive effect against collagen-induced arthritis (CIA), and a peptide *RANK* antagonist was shown to inhibit bone loss [12,13]. Bisphosphonates and zoledronic acid reportedly inhibited osteoclast differentiation [14,15].

Probiotics are well-known to improve the intestinal environment and recently, their beneficial effects on human health are extended to improve various diseases, for instance, type 2 diabetes, non-alcoholic fatty acid disease, and atopic dermatitis [16,17]. The beneficial effects of probiotics on the immune system and gut-bone axis have been recently reported, and probiotics have received considerable attention for treating autoimmune diseases such as RA [18,19]. Among the diverse probiotics, *Lactobacillus* and *Bifidobacterium* have widely been studied in various pathophysiological conditions [20,21]. *Lactobacillus* strains are effective in improving RA by increasing IL-10 production, regulating Th17 and B cell differentiation, reducing pro-inflammatory cytokines, and rebalancing gut microbiota [22,23,24].

Dairy *Propionibacterium freudenreichii* has been used as a cheese starter, and its probiotic and prebiotic properties have been revealed, including anti-inflammatory and bifidogenic activities [25,26]. Previously, we isolated a *P. freudenreichii* strain (designated as MJ2) from raw milk obtained from a local dairy farm and found that heat-killed *P. freudenreichii* promoted osteoblast differentiation and alleviated osteoporosis by increasing the *OPG*/*RANKL* ratio [27]. Therefore, based on our previous findings, we hypothesized that *P. freudenreichii* MJ2 could inhibit osteoclast differentiation, which contributes to the amelioration of RA. In the present study, we examined the inhibitory effect of dairy *P. freudenreichii* MJ2 on osteoclast differentiation and the improvement of CIA in an animal model.

## 2. Materials and Methods

### 2.1. Materials

The murine macrophage cell line, RAW 264.7, was purchased from the Korea Cell Line Bank (KCLB, Seoul, Korea). Dulbecco’s modified Eagle medium (DMEM), minimum essential medium-α modification (α-MEM), fetal bovine serum (FBS), and penicillin/streptomycin were purchased from Hyclone (Logan, UT, USA). MTT (3-[4,5-dimethylthiazol-2-yl]-2,5-diphenyltetrazolium bromide) was obtained from Amresco (Solon, OH, USA), and dimethyl sulfoxide (DMSO) was purchased from Daejung (Siheung, Korea). Recombinant murine *RANK* ligand (*RANKL*) was purchased from Peprotech (Rocky Hill, NJ, USA).

### 2.2. Preparation of Heat-Killed Propionibacterium freudenreichii MJ2 (hkMJ2)

*P. freudenreichii* MJ2 was grown in reinforced clostridial medium (RCM) at 30 °C for 48 h using an anaerobic conditioned chamber (GasPak™ EZ container system; BD, Franklin Lakes, NJ, USA). Bacterial cells were harvested by centrifugation at 3000× *g* for 10 min, and the pellet was washed twice with phosphate-buffered saline (PBS). After diluting bacterial cells to 1 × 10^9^ CFU/mL, live cells were killed by heating at 100 °C for 30 min. The heat-killed *P. freudenreichii* MJ2 (hkMJ2) showed no growth.

### 2.3. Cell Culture and Cytotoxicity Assay

RAW 264.7 cells were grown in DMEM with 10% FBS, 100 U/mL penicillin, and 100 μg/mL streptomycin at 37 °C under 5% CO_2_. The cells were then seeded in 96-well plates at a density of 1 × 10^4^ cells/mL. After 24 h, cells were treated with hkMJ2 (1 × 10^5^, 1 × 10^6^, and 1 × 10^7^ cells/mL) for 4 days. The medium was aspirated and an MTT reagent (0.5 mg/mL) was added. After incubation at 37 °C for 1 h, the supernatant was removed, and DMSO was added to dissolve the formazan crystals. Cell viability was measured at 540 nm using a SpectraMax 340PC384 plate reader (Molecular Devices, Sunnyvale, CA, USA) and calculated as a percentage relative to the negative control group.

### 2.4. Tartrate-Resistant Acid Phosphatase (TRAP) Staining and Activity Assay

RAW 264.7 cells were seeded in 24-well plates at a density of 1 × 10^4^ cells/mL. After overnight incubation at 37 °C, the medium was replaced with α-MEM to differentiate into osteoclasts. Then, cells were treated with *RANKL* (50 ng/mL) and hkMJ2 (1 × 10^5^, 1 × 10^6^, and 1 × 10^7^ cells/mL) for 4 days. Next, cells were washed, fixed, and stained using a TRAP staining kit (Takara Biotechnology, Shiga, Japan), according to the manufacturer’s protocol. TRAP-positive (TRAP(+)) and multinucleated cells were counted under a microscope. TRAP activity was quantified as described previously [28]. Briefly, the fixed cells were incubated with 50 mM citrate buffer (pH 4.5) containing 10 mM sodium tartrate and 6 mM *p*-nitrophenylphosphate for 1 h. The reaction was stopped by adding an equal volume of 0.1 N NaOH solution, and the optical density was measured at 405 nm.

### 2.5. Quantitative Real-Time Polymerase Chain Reaction (qPCR)

RAW 264.7 cells were seeded in 24-well plates at a density of 1 × 10^4^ cells/mL. After overnight incubation at 37 °C, the medium was replaced with α-MEM to differentiate into osteoclasts. Then, cells were treated with *RANKL* (50 ng/mL) and hkMJ2 (1 × 10^5^, 1 × 10^6^, and 1 × 10^7^ cells/mL) for 3 days. Total RNA was extracted with Ribo-Ex reagent (GeneAll Biotechnology, Seoul, Korea), and the amount of total RNA was quantified using a NanoDrop ND-1000 Spectrophotometer (Thermo Scientific, Waltham, MA, USA). After conversion to cDNA with a RevertAid First Strand cDNA Synthesis kit (Thermo Scientific), qPCR was performed using the 7500 Fast Real-Time PCR system (Applied Biosystems, Foster City, CA, USA) using a Kapa SYBR Fast qPCR kit (Kapa Biosystems, Woburn, MA, USA). The reaction was preheated to 95 °C for 10 min, followed by 40 cycles at 95 °C for 15 s, 60 °C for 15 s, and 72 °C for 30 s. Glyceraldehyde-3-phosphate dehydrogenase (*GAPDH*) was used as the reference gene; the primer sequences used in this study are shown in Table 1. Relative gene expression was quantified based on equal amounts of RNA, and the ΔCt (ΔCt = Ct_target gene_ − Ct_reference gene_) value was calculated. The ΔΔCt value was calculated using the following equation: ΔΔCt = (ΔCt_treated_ − ΔCt_untreated_). The normalized expression change was expressed as 2^−ΔΔCt^ (*GAPDH* control was set to 1) [29]. The knee joint was excised, split into small pieces, and homogenized in Ribo-Ex reagent to extract mouse RNA. After centrifugation at 12,000× *g* for 10 min, total RNA was extracted from the supernatant. The qPCR process was the same as that described above.

### 2.6. Western Blotting

RAW 264.7 cells were seeded in 24-well plates at a density of 1 × 10^4^ cells/mL. After overnight incubation at 37 °C, the medium was replaced with α-MEM for cell differentiation into osteoclasts. The cells were treated with *RANKL* (50 ng/mL) and hkMJ2 (1 × 10^5^, 1 × 10^6^, and 1 × 10^7^ cells/mL) for 3 days. Then, cells were harvested, and total protein was extracted using RIPA buffer (Rockland Immunochemicals, Limerick, PA, USA) containing a Halt™ protease inhibitor cocktail (Thermo Scientific). Equal amounts of protein (10–20 μg) quantified by the Bradford assay were denatured and separated by 10% sodium dodecyl sulfate-polyacrylamide gel electrophoresis (SDS-PAGE). The proteins were transferred to PVDF membranes (Millipore, Bedford, MA, USA) and blocked with 5% dry nonfat skim milk in Tris-buffered saline with 0.05% Tween 20 (TBST) for 2 h. After washing with TBST three times, the membrane was incubated with a primary antibody at 4 °C overnight. Antibodies against the endogenous control β-actin (1:5000 dilution, MA5-15739; Thermo Scientific), *RANK* (1:500 dilution, sc-374360; Santa Cruz), phosphorylated p65 *NF-κB* (1:1000 dilution, S536; Cell Signaling, Danvers, MA, USA), p65 *NF-κB* (1:1000 dilution, 8242S; Cell Signaling), *c-fos* (1:1000 dilution, 2250S; Cell Signaling), and *NFATc1* (1:1000 dilution, BD556602; BD Biosciences, Franklin Lakes, NJ, USA) were used. After washing with TBST three times, the membrane was incubated with a secondary antibody for 1 h at room temperature (RT). A goat anti-mouse IgG (H+L) horseradish peroxidase (HRP)-conjugated antibody (1:10,000 dilution, NCI1430KR; Thermo Scientific) was used for anti-β-actin, *RANK*, and *NFATc1* detection, and a goat anti-rabbit IgG (H+L) horseradish peroxidase-conjugated antibody (1:5000 dilution, NCI1460KR; Thermo Scientific) was used to detect other proteins. After washing with TBST, the membrane was developed using a SuperSignal West Femto Maximum Sensitivity Substrate kit (Thermo Scientific). Images were captured and quantified using the ImageJ software (National Institutes of Health, Bethesda, MD, USA).

### 2.7. Animal Model of Collagen-Induced Arthritis (CIA) and Scoring of Severity

Male DBA/1J mice (8 weeks of age) weighing 19–20 g were purchased from Central Lab Animal Inc. (Seoul, Korea). They were maintained at 22 ± 1 °C with a 12 h light/dark cycle at 50 ± 5% humidity. All experimental procedures were approved by the Korea University Institutional Animal Care and Use Committee (approval no. KUIACUC-2020-0025, 11/March, 2020). All experimental procedures were performed according to the Guide for the Care and Use of Laboratory Animals (NIH Publication No. 85-23, 1996). All the mice had free access to food and water. A total of 48 mice were acclimated for 7 days and randomly divided into 6 groups (*n* = 8 per group): normal control group (control), CIA model group (model), CIA mice treated with a high dose of live MJ2 (1 × 10^8^ CFU/mL) group (HLMJ2), CIA mice treated with a low dose of live MJ2 (1 × 10^7^ CFU/mL) group (LLMJ2), CIA mice treated with a high dose of dead MJ2 (1 × 10^8^ cells/mL) group (HDMJ2), and CIA mice treated with a low dose of dead MJ2 (1 × 10^7^ cells/mL) group (LDMJ2) (Table 2). Before CIA induction, the mice were administered live or dead MJ2 daily for 3 weeks, and the administration was continued until the end of the experiment. For CIA induction, an emulsion of 100 μg of bovine type II collagen (CII) (Chondrex, Redmond, WA, USA) in an equal volume of complete Freund’s adjuvant (CFA; Chondrex) was intradermally injected into the tail. After 21 days, a booster of an emulsion of 100 μg CII in incomplete Freund’s adjuvant (IFA; Chondrex) was injected at the same tail site. To evaluate the severity of CIA, the condition of the four paws was monitored and estimated every 4 days on a scale of 0 to 4, as shown in Table 3. The scores of the four paws were summed as arthritic scores.

### 2.8. Micro-Computed Tomography (CT)

Knee joints and paws were fixed in 10% neutral buffered formalin for 48 h and washed with PBS. High-quality 3-dimensional (3D) images of the mouse knee joint and paw were obtained using a SkyScan 1173 micro-CT system (ver 1.6, Konitch, Belgium). Micro-CT was performed on a 1.0 mm region at a resolution of 20 μm. The source voltage was 90 kV, and the source current was 88 μA. Nrecon (ver 1. 7. 4. 2) reconstruction program was used at 20-μm pixel size. Bone parameters, including bone surface/bone volume (BS/TV), bone mineral density (BMD), trabecular thickness (Tb.Th), trabecular number (Tb.N), trabecular separation (Tb.sp), and bone volume/tissue volume (BV/TV), were evaluated.

### 2.9. Histological Analysis

For histological analysis, the hind legs of mice were fixed in 10% neutral buffered formalin, demineralized in 10% ethylenediaminetetraacetate (EDTA), and embedded in paraffin blocks. Paraffin-embedded joint tissues were sectioned and stained with hematoxylin and eosin (H&E). TRAP staining was performed to observe osteoclast differentiation. The sections were observed under a DM750 reverse-phase microscope (Leica Microsystems, Wetzlar, Germany).

### 2.10. Measurement of Cytokines and Collagen-Specific IgG Levels

Whole blood was collected from the sacrificed mice and centrifuged at 3000× *g* and 4 °C for 20 min to obtain serum. The supernatant was collected, and interleukin (IL)-6, TNF-α, and IL-10 levels were measured using an ELISA kit (Thermo Scientific) according to the manufacturer’s instructions. In addition, the levels of total type II collagen-specific IgG (CII-IgG), CII-IgG1, and CII-IgG2a were measured as previously described [30]. In brief, the immunoplate was coated with CII (5 mg/mL) (Chondrex) and incubated at 4 °C overnight. The plate was blocked with 1% bovine serum albumin, and 100 μL of each serum sample was added. After incubation at RT for 2 h, the plate was washed five times with PBS containing 0.05% Tween 20 (PBST) and incubated with biotin-conjugated anti-mouse total IgG antibody (1:250 dilution; 88-50400, Thermo Scientific), HRP-conjugated anti-mouse IgG1 antibody (1:250; 88-50410; Thermo Scientific), and HRP-conjugated anti-mouse IgG2a antibody (1:250; 88-50420, Thermo Scientific) at RT for 1 h. To detect the biotin-conjugated antibody, the plate was subsequently incubated with the avidin-HRP solution at RT for 30 min. After five washes, tetramethylbenzidine (TMB) solution was added for 15 min, and the reaction was stopped by adding 1 N HCl solution. The absorbance was measured at 450 nm, and the relative concentration was calculated and compared with the control group.

### 2.11. Statistical Analysis

In vitro experimental values are expressed as the mean ± standard deviation (SD), and in vivo experimental values are expressed as the mean ± standard error of the mean (SEM). Statistical analyses were performed using SPSS 24.0 (IBM Corp., Armonk, NY, USA). Differences between any two groups were examined using the Student’s *t*-test, and differences among groups were determined by one-way analysis of variance (ANOVA), followed by Tukey’s honestly significant difference (HSD) post-hoc test. Statistical significance was set at *p* < 0.05.

## 3. Results

### 3.1. Cytotoxicity of hkMJ2 in RAW 264.7 Cells

**Figure 1:**
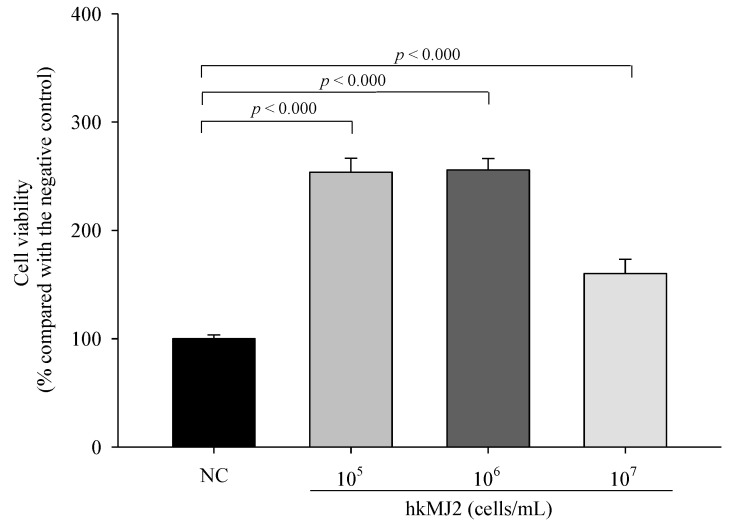
Cytotoxicity of hkMJ2 against RAW 264.7 cells. The values indicate the mean ± standard deviation (SD) of three independent experiments performed in triplicate. A Student’s t-test was used to determine the significance of the difference.

RAW 264.7 cells, a murine macrophage cell line, are known to readily differentiate into osteoclast following *RANKL* exposure [31], and thus RAW 264.7 cells were used to investigate the inhibitory effect of hkMJ2 on osteoclast differentiation. The MTT assay was employed to determine the cytotoxicity of hkMJ2 against RAW 264.7. We observed that cells treated with hkMJ2 (1 × 10^5^, 1 × 10^6^, and 1 × 10^7^ cells/mL) showed a significant increase in viability (Figure 1). Therefore, we used hkMJ2 at the concentrations in the following experiments.

### 3.2. HkMJ2 Inhibits Osteoclastogenesis and TRAP Activity in Raw 264.7 Cells

**Figure 2:**
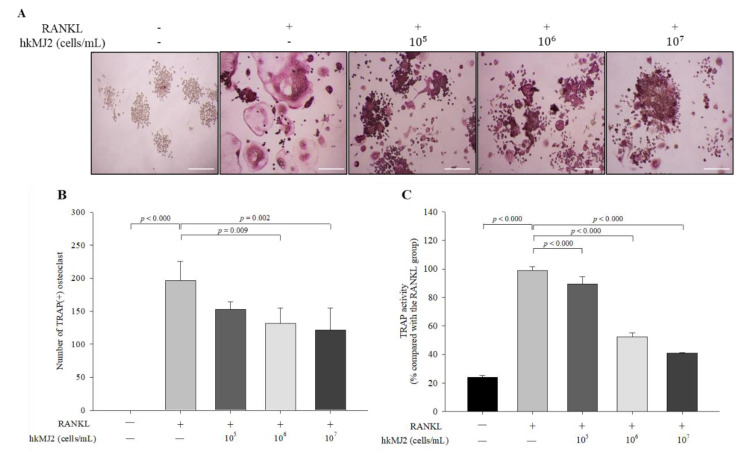
Effect of hkMJ2 on TRAP(+) number and TRAP activity. RANKL-induced osteoclast formation was measured by TRAP staining (**A**) (100×, scale bar = 100 μm), and the number of TRAP(+) multinucleated osteoclasts were counted (**B**), and TRAP activity was quantified (**C**). The values indicate the mean ± standard deviation (SD) of three independent experiments performed in triplicate. *RANKL*, receptor activator of the nuclear factor-κB ligand; TRAP, tartrate-resistant acid phosphatase. −, no addition; +, addition. One-way ANOVA followed by Tukey’s HSD was used to determine significance.

To investigate the inhibitory effect of hkMJ2 on osteoclast formation, TRAP(+) multinucleated osteoclast differentiation was measured using TRAP staining and activity assay. Although the number of TRAP(+) osteoclasts in the 1 × 10^5^ cells/mL of hkMJ2-treated cells did not significantly decrease, 1 × 10^6^ and 1 × 10^7^ cells/mL hkMJ2 treatment showed a significant inhibitory effect on TRAP(+) osteoclast formation (Figure 2A,B). TRAP activity significantly (*p* < 0.000) decreased in the hkMJ2-treated cells in a dose-dependent manner when compared with the RANKL-only treated group (Figure 2C). Osteoclasts are TRAP(+) multinucleated cells that show high TRAP expression. TRAP participates in osteoclast-mediated bone turnover; thus, overexpression of TRAP is associated with increased bone turnover [32]. TRAP staining showed that hkMJ2 decreased the formation of mature osteoclasts, indicating that hkMJ2 inhibits osteoclastogenesis and TRAP activity.

### 3.3. HkMJ2 Decreases the Expression Levels of Osteoclast-Related Genes and Proteins

**Figure 3:**
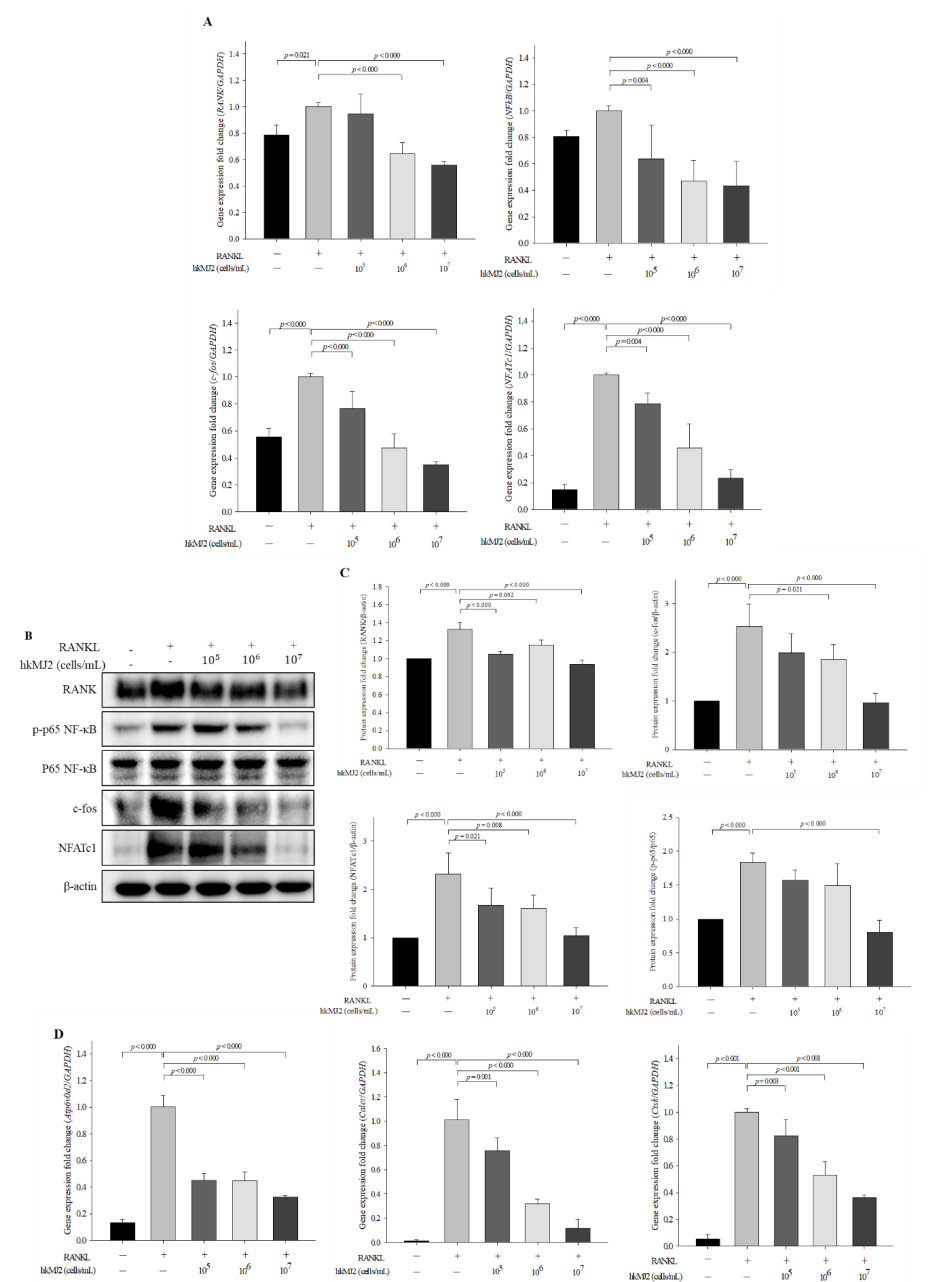
Effects of hkMJ2 on the expression levels of osteoclast differentiation-related genes and proteins in the RANKL-induced cells. The expression levels of RANKL-induced osteoclastogenic genes were measured by qPCR (**A**) and proteins were measured by western blotting (**B**) and quantified (**C**). The expression levels of *NFATc1*-downstream genes were measured by qPCR (**D**). The values indicate the mean ± standard deviation (SD) of three independent experiments performed in triplicate. *NFATc1*, nuclear factor of activated T-cells cytoplasmic 1; *RANKL*, receptor activator of nuclear factor-κB ligand; *RANK*, receptor activator of nuclear factor-κB; *NF-κB*, nuclear factor kappa light chain enhancer of activated B cells; *Atp6v0d2*, V-type proton ATPase subunit d2; *Calcr*, calcitonin receptor; *Ctsk*, cathepsin K. −, no addition; +, addition. One way ANOVA followed by Tukey’s HSD was used to determine significance.

To investigate the effect of hkMJ2 on the expression levels of genes and proteins related to osteoclast differentiation, we performed qPCR and western blotting, respectively. *RANK* expression was significantly decreased in the cells treated with 1 × 10^6^ and 1 × 10^7^ cells/mL hkMJ2 when compared with those treated with *RANKL* alone (Figure 3A). In addition, the expression levels of RANKL-induced osteoclastogenic genes, including nuclear factor kappa light chain enhancer of activated B cells (*NF-κB*), *c-fos*, and nuclear factor of activated T-cells cytoplasmic 1 (*NFATc1*), were significantly decreased in hkMJ2-treated cells in a dose-dependent manner when compared with those treated with *RANKL* alone. At the protein level, the expression levels of *RANK* and *NFATc1* were significantly reduced in hkMJ2-treated cells in a dose-dependent manner when compared with those treated with *RANKL* alone (Figure 3B,C). The level of activated *NF-κB* significantly (*p* < 0.000) decreased in the cells treated with 1 × 10^7^ cells/mL hkMJ2 and the level of *c-fos* significantly decreased in the cells treated with 1 × 10^6^ and 1 × 10^7^ cells/mL hkMJ2 when compared with those treated with *RANKL* alone. Following hkMJ2 treatment, expression levels of *NFATc1*-downstream genes such as V-type proton ATPase subunit d2 (*Atp6v0d2*), calcitonin receptor (*Calcr*), and cathepsin K (*Ctsk*), significantly decreased in a dose-dependent manner (Figure 3D). The results suggest that hkMJ2 inhibits RANKL-induced osteoclast differentiation by inhibiting the *NF-κB* signaling pathway.

### 3.4. MJ2 Attenuates Collagen-Induced Arthritis (CIA)-Associated Symptoms

**Figure 4:**
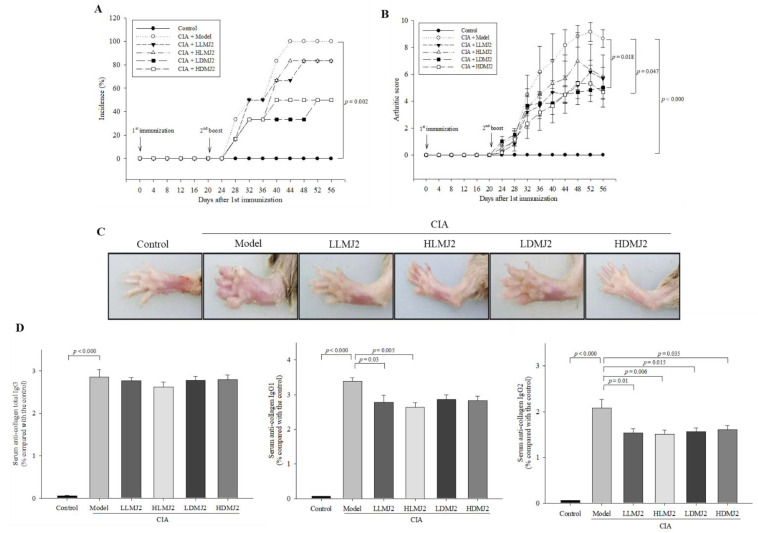
Effects of MJ2 on collagen-induced arthritis (CIA)-associated symptoms (*n* = 8 per group). Incidence of arthritis (**A**), rheumatic clinical score (**B**), and representative images of mice paw thickness (**C**). Levels of collagen-specific IgG antibodies were measured by ELISA (**D**). Data values indicate the mean ± standard error of the mean (SEM). One way ANOVA followed by Tukey’s HSD was used to determine significance.

To evaluate the ameliorative effect of MJ2 on RA in vivo, a CIA mouse model was established. MJ2 treatment did not significantly affect the incidence of arthritis, however, MJ2 treatment showed a decreasing trend in the incidence of arthritis (Figure 4A). On the other hand, oral administration of dead MJ2 significantly decreased the arthritis score when compared with the model group, while although live MJ2 showed a reduced arthritis score, it failed to demonstrate a significant suppressive effect (Figure 4B,C). In CIA model mice, the most commonly used arthritis model, live and dead MJ2 administration alleviated arthritis, although it did not significantly reduce the incidence of arthritis.

Although total IgG levels were unaltered, the serum CII-specific IgG1 levels in HLMJ2 and LLMJ2 groups significantly decreased, and the serum CII-specific IgG2a levels of all MJ2-administrated groups significantly decreased when compared with the model group (Figure 4D). These results suggested that MJ2 administration can ameliorate arthritis by decreasing the level of collagen-specific IgG antibodies.

### 3.5. MJ2 Inhibits the Bone Erosion in CIA Mice

**Figure 5:**
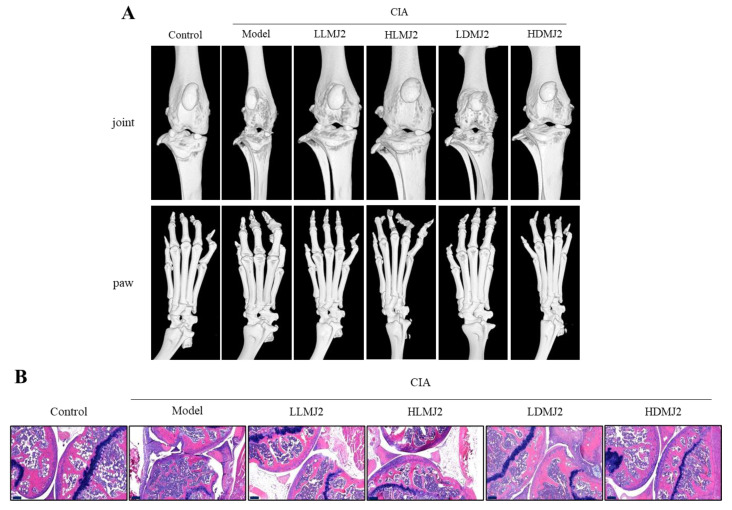
Ameliorative effect of MJ2 on bone erosion in CIA mice (*n* = 8 per group). Mice knee joint and paw were observed by micro-CT. Representative images of mice knee and paw (**A**). The inflammation in the mice knee joint was observed by hematoxylin-eosin staining (**B**) (100×, scale bar = 200 μm). Data values indicate the mean ± standard error of the mean (SEM). One way ANOVA followed by Tukey’s HSD was used to determine significance. CIA, collage-induced arthritis.

Micro-CT was performed to evaluate the degree of distortion and erosion of the knee and plantar bones. Although no significant difference in the plantar bone distortion was observed, the knee bone joint in the model group was distinctly damaged when compared with the normal control, and those of the MJ2-administrated groups were reduced the joint destruction when compared with the model group (Figure 5A). administration, Especially in the HDMJ2 group, BS/TV, BMD, BV/TV, Tb.Th, and Tb.N significantly increased and Tb.sp significantly decreased when compared with the model group (Table 4). BMD significantly increased in all MJ2-treated groups except HLMJ2 and Tb.Th significantly increased in all MJ2-treated groups compared with the model group. Interestingly, the results showed that dead MJ2 was more effective than live MJ2 in the CIA in vivo model.

Histopathological analysis of the knee joint section revealed that tissue damage and bone erosion deteriorated in the model group when compared with the NC group. However, the MJ2-administered groups showed ameliorated bone erosion, leukocyte infiltration, and cartilage damage when compared with the model group (Figure 5B). These results suggested that MJ2 administration inhibits bone loss and affords protection against bone erosion and joint damage in a CIA mouse model.

### 3.6. MJ2 Decreases the Levels of Proinflammatory Cytokines and Increases the Level of IL-10 in CIA Mice

**Figure 6:**
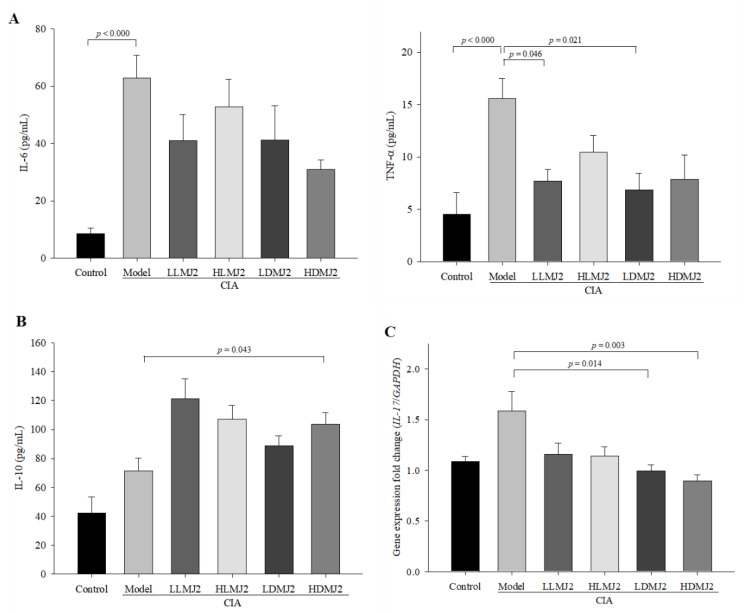
Effect of MJ2 on the expression levels of pro-inflammatory and anti-inflammatory cytokines in CIA mice (*n* = 8 per group). Protein levels of pro-inflammatory (**A**) and anti-inflammatory (**B**) cytokines were measured in mice serum by ELISA. Gene expression levels of *IL-17* were measured in the knee joint of mice by qPCR (**C**). Data values indicate the mean ± standard error of the mean (SEM). One way ANOVA followed by Tukey’s HSD was used to determine significance. CIA, collagen-induced arthritis; IL, interleukin; TNF-α, tumor necrosis factor-α.

We next evaluated the effect of MJ2 on CIA-mediated pro-inflammatory and anti-inflammatory cytokine production. Accordingly, we measured the cytokine levels in mouse serum using ELISA. The expression levels of typical pro-inflammatory cytokines, IL-6 and TNF-α, were significantly (*p* < 0.000) elevated following CIA induction, and MJ2 administration typically reduced the levels of pro-inflammatory cytokines (Figure 6A). Although the level of IL-6 did not significantly decrease in the MJ2-administrated group, the expression level of TNF-α was significantly reduced in the LLMJ2 (*p* = 0.046) and LDMJ2 (*p* = 0.021) groups when compared with the model group. Conversely, the expression level of the anti-inflammatory cytokine, IL-10, was increased in the MJ2-administered groups, although only the HDMJ2 group displayed statistical significance when compared with the model group (Figure 6B). The expression level of *IL-17*, which leads to the worsening of RA, was significantly decreased in the LDMJ2 (*p* = 0.014) and HDMJ2 (*p* = 0.003) groups when compared with the model group (Figure 6C). These results suggested that MJ2 improves inflammation by decreasing pro-inflammatory cytokines and increasing IL-10 levels.

### 3.7. MJ2 Inhibits the Osteoclast Differentiation in CIA Mice

**Figure 7:**
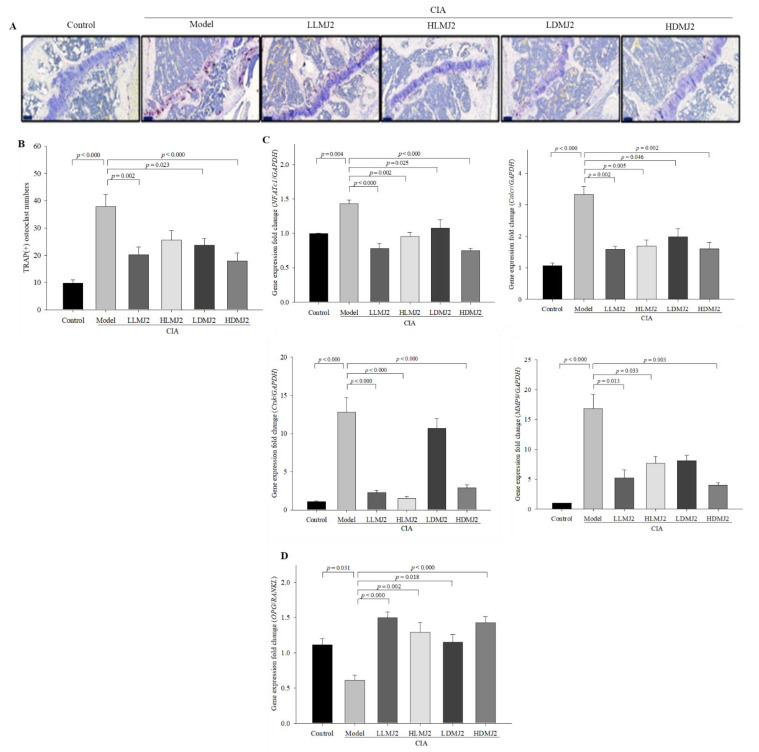
Inhibitory effect of MJ2 on osteoclast differentiation in CIA mice (*n* = 8 per group). Osteoclasts were stained with TRAP staining in mice knee joints (100×, scale bar = 100 μm) (**A**), and TRAP(+) cells were counted (**B**). Expression levels of osteoclast differentiation-related genes (**C**) and *OPG*/*RANKL* ratio (**D**) were measured by qPCR. Data values indicate the mean ± standard error of the mean (SEM). One-way ANOVA followed by Tukey’s HSD was used to determine significance. CIA, collagen-induced arthritis; TRAP, tartrate-resistant acid phosphatase; *NFATc1*, nuclear factor of activated T-cells cytoplasmic 1; *Calcr*, calcitonin receptor; *Ctsk*, cathepsin K; MMP-9, matrix metalloproteinase-9; *RANKL*, receptor activator of the nuclear factor-κB ligand; *OPG*, osteoprotegerin.

TRAP staining was performed to evaluate the effect of MJ2 on osteoclast differentiation in the CIA model. TRAP(+) osteoclasts were observed at the erosion front within the synovium, and the number of stained cells was significantly higher in the model group (*p* < 0.000) than that in the normal control. In addition, TRAP(+) osteoclasts were significantly decreased in the MJ2-administrated groups, except in the HLMJ2 group, compared with the model group (Figure 7A,B). TRAP-positive multinucleated cells are known to be present in the synovium at sites of cartilage destruction in patients with RA [33]. Furthermore, TRAP-positive multinucleated cells produce MMP-2 (matrix metalloproteinase-2) and MMP-9, which may contribute to cartilage destruction of bone in patients with RA.

Among the osteoclast differentiation-related genes, expression levels of *NFATc1* and *Calcr* were significantly reduced in the MJ2-administered groups and expression levels of *Ctsk* and *MMP9* were significantly reduced in the MJ2-administered groups except for the LDMJ2 group when compared with the model group (Figure 7C). Meanwhile, the *OPG*/*RANKL* ratio, an indicator of inhibition of osteoclast differentiation, was significantly increased by MJ2 administration (Figure 7D). *P. freudenreichii* MJ2 reportedly increases osteoblast differentiation and BMD by enhancing the *OPG*/*RANKL* ratio [27]. In the present study, the *OPG*/*RANKL* ratio was significantly increased in MJ2 administered groups, which might contribute to an increase in BMD by inhibiting osteoclast differentiation. These results consisted of the results of BMD in the MJ2-administered groups except the HLMJ2 group in Table 4. These results suggested that MJ2 administration inhibits osteoclast differentiation and TRAP activity in the CIA mouse model.

## 4. Discussion

Probiotics are useful bacteria that improve human health, and they are being studied in many different fields. Among them, *Lactobacillus* and *Bifidobacterium* species are the main probiotics that have been extensively studied [34,35,36]. *Lactobacilli* show a protective effect against CIA and interestingly, their ability to alleviate RA are different according to the species [37]. *L. casei*, *L. rhamnosus*, and *L. fermentum* protect RA through modulating immune response and rebalancing gut microbiota, while *L. salivarius* delays the development of RA without affecting immune response. A probiotic *L. casei* shows a protective effect against gut dysbiosis and bone destruction in complete Freund’s adjuvant (CFA)-induced RA [38]. The gut-joint axis has been studied to apply for the treatment of RA, which means that modulation of the gut microbiota might be useful to prevent or treat RA [39]. The alteration of the gut microbiome and a perturbation of metabolites involved in energy production and the metabolism of fatty acid and secondary bile acid could contribute to the development of RA. In this study, *P. freudenreichii* MJ2 improves RA and protects bone destruction. In a further study, we need to analyze the gut microbiome to confirm that administration of *P. freudenreichii* MJ2 restores gut dysbiosis in the CIA model. Beneficial gut microbiota produces short-chain fatty acids (SCFAs) such as butyrate, propionate, and acetate that serve as substrates for other gut microbial members. *Lactobacillus* and *Bifidobacterium* produce SCFAs, which contribute to preventing gut dysbiosis. Increased levels of SCFAs in RA patients administered a high-fiber diet decrease pro-inflammatory cytokines and modulate gut microbiota composition, which contributes to improving RA [40]. SCFA supplementation directly or through a high-fiber diet protects bone loss [41] and shows beneficial effects on bone homeostasis in RA patients [42]. Oral administration of *P. freudenreichii* MJ2 settles in the gut and increases the level of propionic acid in the feces of the rats with dextran sodium sulfate (DSS)-induced colitis [43]. It suggests that propionic acid produced by *P. freudenreichii* MJ2 may contribute to improving CIA.

RA is a typical disease that occurs in all age groups; however, the exact cause is still unknown [1]. Since RA is characterized by continuous inflammation in the joints, it leads to pain and further bone destruction [44]. Osteoclasts are the major cells that destroy the bone matrix and exacerbate inflammation in RA [7]. Thus, inhibition of osteoclast differentiation is an emerging new target to treat RA [10]. Osteoclasts are multinucleated cells that are differentiated from monocyte-macrophage lineage. *RANKL* is a key factor of osteoclast differentiation and bone destruction in RA. In the process of osteoclast differentiation, *RANKL* stimulates the adhesion of osteoclast precursor cells to the bone matrix, and then, activated immature osteoclasts gradually differentiate into a mature state [10]. Osteoclasts attach to the bone surface matrix, create an acidic environment, and secrete osteoclast differentiation-related factors, including TRAP, *Ctsk*, and *Calcr*, which mediate bone destruction [45]. *L. reuteri* 6475 and its metabolite, lactobacillic acid prevent osteoclastogenesis via binding to a long-chain fatty acid receptor, GPR120, on the surface of RAW264.7 cells [46]. *L. rhamnosus* prevents bone loss through inhibiting osteoclastogenesis in RANKL-induced osteoclast differentiation and through immunomodulation in ovariectomy mice [47]. *P. freudenreichii* MJ2 strain isolated from raw milk shows anti-inflammatory activity and improves bone health by promoting osteoblast differentiation [27]. In this study, we found that *P. freudenreichii* MJ2 inhibited osteoclast differentiation in RANKL-induced in vitro model. *P. freudenreichii* MJ2 inhibited the expression levels of *NFATc1*, which is a master transcription regulator of osteoclast, and *ctsk*, *calcr*, and atp6vod2 which play a role in bone resorption by osteoclast. In TRAP and f-actin staining, *P. freudenreichii* MJ2 decreased the formation of mature osteoclast. The *NF-κB* signaling pathway is involved in RANKL-induced osteoclast differentiation [48]. *RANKL* stimulates the expression of *NFATc1* through *NF-κB* and *c-Fos* activation [49]. *NFATc1*, a master regulator of osteoclast differentiation, is activated in the early stage of osteoclast differentiation by *c-Fos*, followed by inducing the production of factors involved in bone resorption of osteoclasts, such as TRAP, cathepsin K (*Ctsk*), calcitonin receptor (*Calcr*), and matrix metallopeptidase (MMP)-9. Thus, *NFATc1* and *c-Fos* are key factors for osteoclast differentiation. In the present study, hkMJ2 inhibited the gene expression and activation of *NF-κB*, as well as significantly decreased gene and protein expression levels of *NFATc1* and *c-Fos* and expression levels of *NFATc1*-downstream genes, suggesting that hkMJ2 inhibits RANKL-induced osteoclast differentiation by inhibiting the *NF-κB* signaling pathway.

The collagen-induced arthritis (CIA) model is the most typical in vivo model of arthritis in which CIA susceptible DBA/1 mouse is used [50,51]. *L. salivarius* delays the onset of CIA, while it shows no relief from the severity of CIA [37]. Interestingly, in this study, although it was not effective in the reduction of arthritis incidence, oral administration of *P. freudenreichii* MJ2 alleviated arthritic scores such as swelling of paw and degree of redness. *P. freudenreichii* MJ2 administration decreased the level of collagen-specific IgG2 antibody that indicates the severity of RA. IgG2 aggravates arthritis by recruiting neutrophils and macrophages in the inflamed lesion [52], thus the reduced CII-IgG2 level by *P. freudenreichii* MJ2 might lead to a reduction of inflammatory response resulted in the alleviation of RA severity. Furthermore, *P. freudenreichii* MJ2 decreased the level of TNF-α and *IL-17* that are important indicators of inflammation, while *P. freudenreichii* MJ2 administration increased IL-10, a representative anti-inflammatory cytokine. TNF-α and *IL-17* exacerbate RA by promoting monocyte activation and osteoclastogenesis [53,54]. In addition, *P. freudenreichii* MJ2 administration increased the *OPG*/*RANKL* ratio and inhibited osteoclast activity, which might prevent joint bone deterioration and destruction in the CIA model. These results suggest that *P. freudenreichii* MJ2 alleviated RA symptoms by blocking osteoclast differentiation, enhancing the production of IL-10, and inhibiting the production of pro-inflammatory cytokines. Taken together, *P. freudenreichii* MJ2 alleviates RA symptoms and inflammation without reducing the incidence of RA, thus *P. freudenreichii* MJ2 might alleviate RA symptoms rather than prevent the development of RA itself.

## 5. Conclusions

*P. freudenreichii* MJ2 inhibits osteoclast differentiation by inhibiting the *NF-κB* signaling pathway and preventing CIA and bone destruction. In addition, we observed that both live and dead *P. freudenreichii* MJ2 improved RA in a CIA animal model. Dairy *P. freudenreichii* has been listed as “generally recognized as safe” (GRAS) by the Food and Drug Administration, which implies that *P. freudenreichii* MJ2 is safe. Therefore, our findings suggest that *P. freudenreichii* MJ2 isolated from raw milk could potentially be developed as an agent for alleviating RA. Further studies are necessary to elucidate the effective component(s) and mode of action of *P. freudenreichii* MJ2 on RA amelioration.

## Figures and Tables

**Table 1:** Primer sequences used for qPCR.

Gene	Forward (5′→3′)	Reverse (5′→3′)
*GAPDH*	ACCCAGAAGACTGTGGATGG	CACATTGGGGGTAGGAACAC
*RANK*	TGCAGCTCAACAAGGATACG	GAGCTGCAGACCACATCTGA
*NF-κB*	TCCTGGCCTCTAGCCTTGTA	GCCAAGGAAGAAAAGTGCTG
*c-fos*	CCAGTCAAGAGCATCAGCAA	AAGTAGTGCAGCCCGGAGTA
*NFATc1*	GGTGCTGTCTGGCCATAACT	GCGGAAAGGTGGTATCTCAA
*MMP9*	GAAGGCAAACCCTGTGTGTT	AGAGTACTGCTTGCCCAGGA
*Atp6v0d2*	GACCCTGTGGCACTTTTTGT	GCTTGCATTTGGGGAATCTA
*Calcr*	CGGACTTTGACACAGCAGAA	GTCACCCTCTGGCAGCTAAG
*Ctsk*	CAGCTTCCCCAAGATGTGAT	AGCACCAACGAGAGGAGAAA
*OPG*	CTGCCTGGGAAGAAGATCAG	TTGTGAAGCTGTGCAGGAAC
*RANKL*	AGCCGAGACTACGGCAAGTA	GCGCTCGAAAGTACAGGAAC
*IL-17*	TGAGTCCAGGGAGAGCTTCA	TTCATTGCGGTGGAGAGTCC

**Table 2:** Animal groups were used in this study.

Group	Treatment	CIA Induction
Normal control	PBS	−
Model	PBS	+
LLMJ2	Low-dose live *P. freudenreichii* MJ2 (1 × 10^7^ CFU/mL)	+
HLMJ2	High-dose live *P. freudenreichii* MJ2 (1 × 10^8^ CFU/mL)	+
LDMJ2	Low-dose dead *P. freudenreichii* MJ2 (1 × 10^7^ cells/mL)	+
HDMJ2	High-dose dead *P. freudenreichii* MJ2 (1 × 10^8^ cells/mL)	+

PBS, phosphate-buffered saline; CIA, collagen-induced arthritis. −, no CIA induction; +, CIA induction.

**Table 3:** Qualitative scoring system to assess the severity of CIA inflammation.

Score	Condition
0	No signs
1	Mild but definite redness and swelling of the ankle or wrist, or apparent redness and swelling limited to individual digits, regardless of the number of affected digits
2	Moderate redness and swelling of ankle or wrist
3	Severe redness and swelling of the entire paw, including digits
4	Maximally inflamed limb with involvement of multiple joints

CIA, collagen-induced arthritis.

**Table 4:** Bone parameters in CIA mice measured by micro-CT (*n* = 8 per group).

Group	BS/TV (1/mm)	BMD (g/cm^3^)	BV/TV (%)	Tb.Th (mm)	Tb.N (1/mm)	Tb.sp (mm)
Control	12.513 ± 0.343 ^###^	0.293 ± 0.007 ^###^	34.293 ± 0.823 ^###^	0.111 ± 0.002 ^###^	3.152 ± 0.077 ^###^	0.184 ± 0.007 ^###^
Model	4.992 ± 0.834 ***	0.131 ± 0.007 ***	8.092 ± 1.118 ***	0.082 ± 0.002 ***	0.976 ± 0.115 ***	0.340 ± 0.022 ***
LLMJ2	7.356 ± 0.864	0.186 ± 0.016 ^#^	13.131 ± 1.185	0.105 ± 0.003 ^##^	1.353 ± 0.162	0.262 ± 0.013 ^#^
HLMJ2	6.369 ± 0.295	0.168 ± 0.009	13.235 ± 1.161	0.098 ± 0.007 ^#^	1.344 ± 0.050	0.275 ± 0.021
LDMJ2	6.500 ± 0.336	0.197 ± 0.008 ^##^	12.792 ± 1.166	0.110 ± 0.001 ^###^	1.373 ± 0.110	0.312 ± 0.016
HDMJ2	8.376 ± 0.956 ^#^	0.193 ± 0.015 ^##^	16.418 ± 3.474 ^#^	0.113 ± 0.002 ^###^	1.930 ± 0.308 ^##^	0.244 ± 0.007 ^##^

One way ANOVA followed by Tukey’s HSD was used to determine significance. *** *p* < 0.001 compared with the control; ^#^ *p* < 0.05, ^##^ *p* < 0.01, ^###^ *p* < 0.001 compared with the model. BMD, bone mineral density; BS/TV, bone surface/bone volume; BV/TV, bone volume/tissue volume; Tb.N, trabecular number; Tb.Th, trabecular thickness; Tb.sp, trabecular separation.

## Data Availability

The information on the data utilized for analysis is provided in the Section 2 of this manuscript.
